# A review on the role of LINC00511 in cancer

**DOI:** 10.3389/fgene.2023.1116445

**Published:** 2023-04-14

**Authors:** Soudeh Ghafouri-Fard, Arash Safarzadeh, Bashdar Mahmud Hussen, Mohammad Taheri, Seyed Abdulmajid Ayatollahi

**Affiliations:** ^1^ Department of Medical Genetics, School of Medicine, Shahid Beheshti University of Medical Sciences, Tehran, Iran; ^2^ Men’s Health and Reproductive Health Research Center, Shahid Beheshti University of Medical Sciences, Tehran, Iran; ^3^ Department of Clinical Analysis, College of Pharmacy, Hawler Medical University, Erbil, Iraq; ^4^ Institute of Human Genetics, Jena University Hospital, Jena, Germany; ^5^ Urology and Nephrology Research Center, Shahid Beheshti University of Medical Sciences, Tehran, Iran; ^6^ Phytochemistry Research Center, Shahid Beheshti University of Medical Sciences, Tehran, Iran

**Keywords:** LINC00511, cancer, biomarker, expression, diagnostic

## Abstract

Long Intergenic Non-Protein Coding RNA 511 (LINC00511) is an RNA gene being mostly associated with lung cancer. Further assessments have shown dysregulation of this lncRNA in a variety of cancers. LINC00511 has interactions with hsa-miR-29b-3p, hsa-miR-765, hsa-mir-150, miR-1231, TFAP2A-AS2, hsa-miR-185-3p, hsa-miR-29b-1-5p, hsa-miR-29c-3p, RAD51-AS1 and EZH2. A number of transcription factors have been identified that regulate expression of LINC00511. The current narrative review summarizes the role of LINC00511 in different cancers with an especial focus on its prognostic impact in human cancers.

## Introduction

Long non-coding RNAs (lncRNAs) are widely expressed transcripts with essential roles in gene regulation. Based on the results of the Human GENCODE project, the number of lncRNA genes in the human genome is estimated to surpass 16,000 (Fang et al., 2018). These transcripts embrace lncRNAs transcribed by RNA polymerase II, gene-overlapping antisense transcripts as well as lncRNAs from intergenic regions (lincRNAs) (Ghafouri-Fard et al., 2020; Ghafouri-Fard et al., 2021; Statello et al., 2021). Typically, lncRNAs have a 7-methyl guanosine cap at the 5′end and a polyadenylated tail at the 3′ends. Moreover, lncRNAs are spliced in a similar manner to mRNAs (Statello et al., 2021). However, several RNA polymerase II-transcribed lncRNAs are incompetently processed and are held in the nuclear compartment (Statello et al., 2021). LncRNAs interact with DNA, RNA and proteins. Through these interactions, lncRNAs influence chromatin structure and function, affect the assembly of nuclear bodies, change the stability and expression of mRNAs within the cytoplasm and regulate signaling pathways (Statello et al., 2021; Hussen et al., 2022). In comparison with mRNA promoters, lincRNA promoters have been found to be devoid of transcription factor binding sites, but having binding sites for a number of specific factors such as GATA and FOS (Melé et al., 2017).

Long Intergenic Non-Protein Coding RNA 511 (LINC00511) is an RNA gene being mostly associated with lung cancer. This lncRNA is encoded on chr17:72,290,091-72,640,472 (GRCh38/hg38), minus strand. Notably, more than 100 alternatively splice variants have been recognized for LINC00511. Except for two variants with retained introns (LINC00511-279 with 3766 bp and LINC00511-278 with 508 bp), other are affiliated with lncRNA group of transcripts (https://asia.ensembl.org/Homo_sapiens/Gene/Summary?db=core;g=ENSG00000227036; r=17:72290091-72640472). This lncRNA has important roles in the development of cancers and can be used as a possible diagnostic and prognostic marker in cancer. Abnormal expression of LINC00511 in a wide array of malignancies potentiates it as a target for therapeutic interventions. Based on DIANA-LncBase database (https://diana.e-ce.uth.gr/lncbasev3) (Karagkouni et al., 2020), expression of LINC00511 in various tissues and cell types have been investigated. With medium and high TPM levels and in *homosapiens*, the highest level of expression of LINC00511 is in prostate tissue and PC3 cell type along with cancer/malignant category.

The current narrative review summarizes the role of LINC00511 in different cancers with an especial focus on its prognostic impact in human cancers.

### LINC00511 in cancers

Several studies have reported dysregulation (mainly upregulation) of LINC00511 in different cancers. These studies have also identified miRNAs that are sponged by LINC00511.

### Breast cancer

**FIGURE 1:**
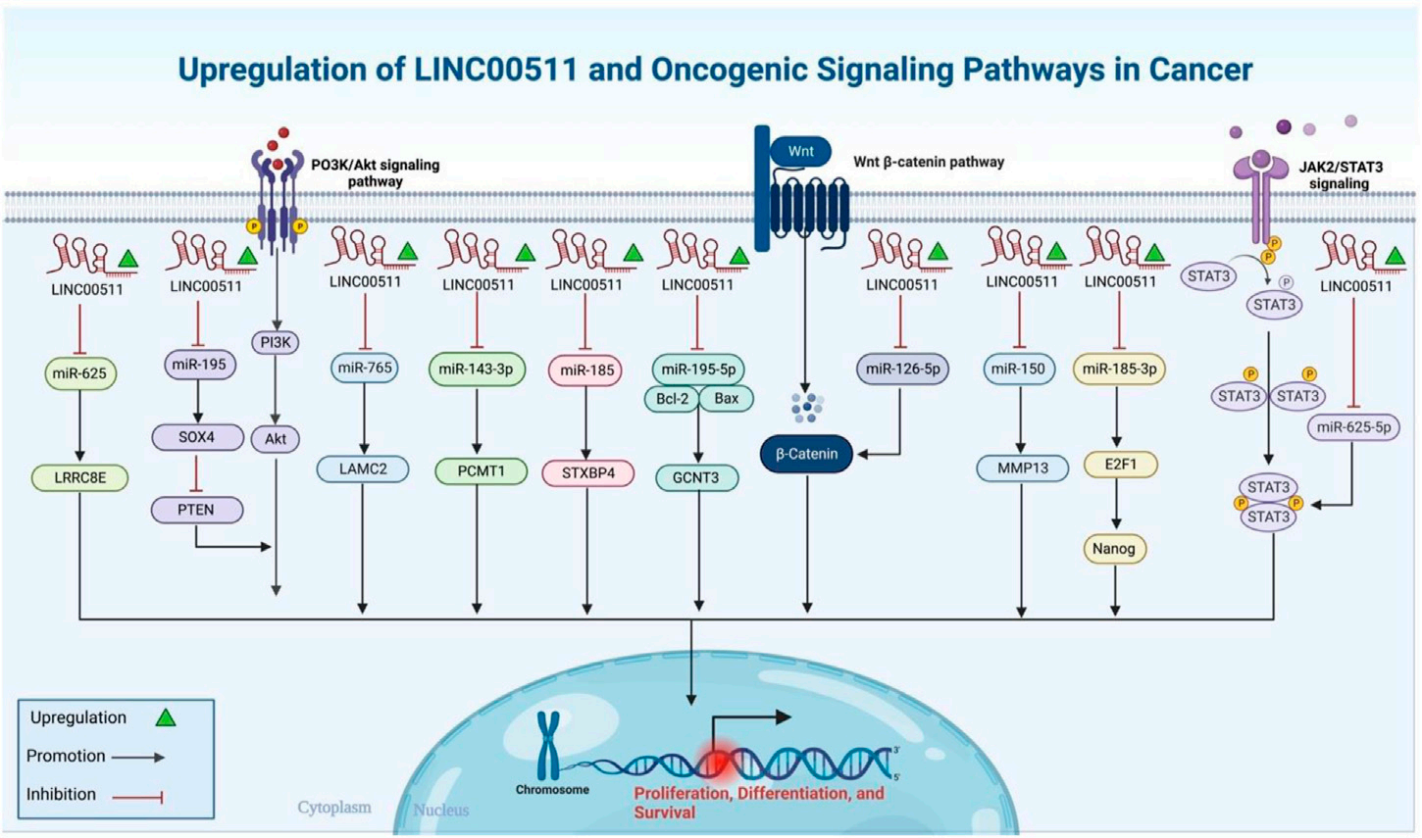
A graphical illustration of the oncogenic role that LINC00511 performs in the environment of the many types of cancer.

In breast cancer, LINC00511 has been found to be highly expressed in the clinical samples and its over-expression has been correlated with poor prognosis (Lu et al., 2018). Functional studies have shown that LINC00511 promotes proliferation, sphere-formation capacity, expression of stem factors and growth of breast tumors (Lu et al., 2018). From a mechanical point of view, LINC00511 acts as a molecular sponge for miR-185-3p to enhance expression of E2F1 protein. Besides, E2F1 binds with the promoter of Nanog gene and increases its expression (Figure 1). Therefore, LINC00511/miR-185-3p/E2F1/Nanog axis has been identified as an important route for induction of stemness and tumorigenesis in breast cancer (Lu et al., 2018). Another study in breast cancer has revealed more than 180 potential targets for LINC00511 through siRNA and RNA-seq assays. Bioinformatics analyses have shown relation between differently expressed genes and signaling pathways mediated by p38-α and p38-β. LINC00511 has been found to be mainly located in the cytoplasm regulating expression of MMP13 through sponging miR-150 (Shi et al., 2021). Expression of LINC00511 in breast cancer samples has been closely correlated with the presence of lymph node metastasis, greater tumor size and molecular subtypes of breast cancer. This lncRNA has been found to increase migratory potential and invasive ability of MDA-MB-231 and MCF-7 cells. Moreover, expression of LINC00511 has been shown to be increased by DNA hypomethylation. In turn, LINC00511 could promote expressions of Wnt10A, E2F2, TGFA, and MET and reduce sensitivity of breast cancer cells to Panobinostat (Liu et al., 2021a). LINC00511 can also influence the cytotoxic effects of paclitaxel on breast cancer cells through regulating miR-29c/CDK6 axis (Zhu et al., 2019).

**FIGURE 2:**
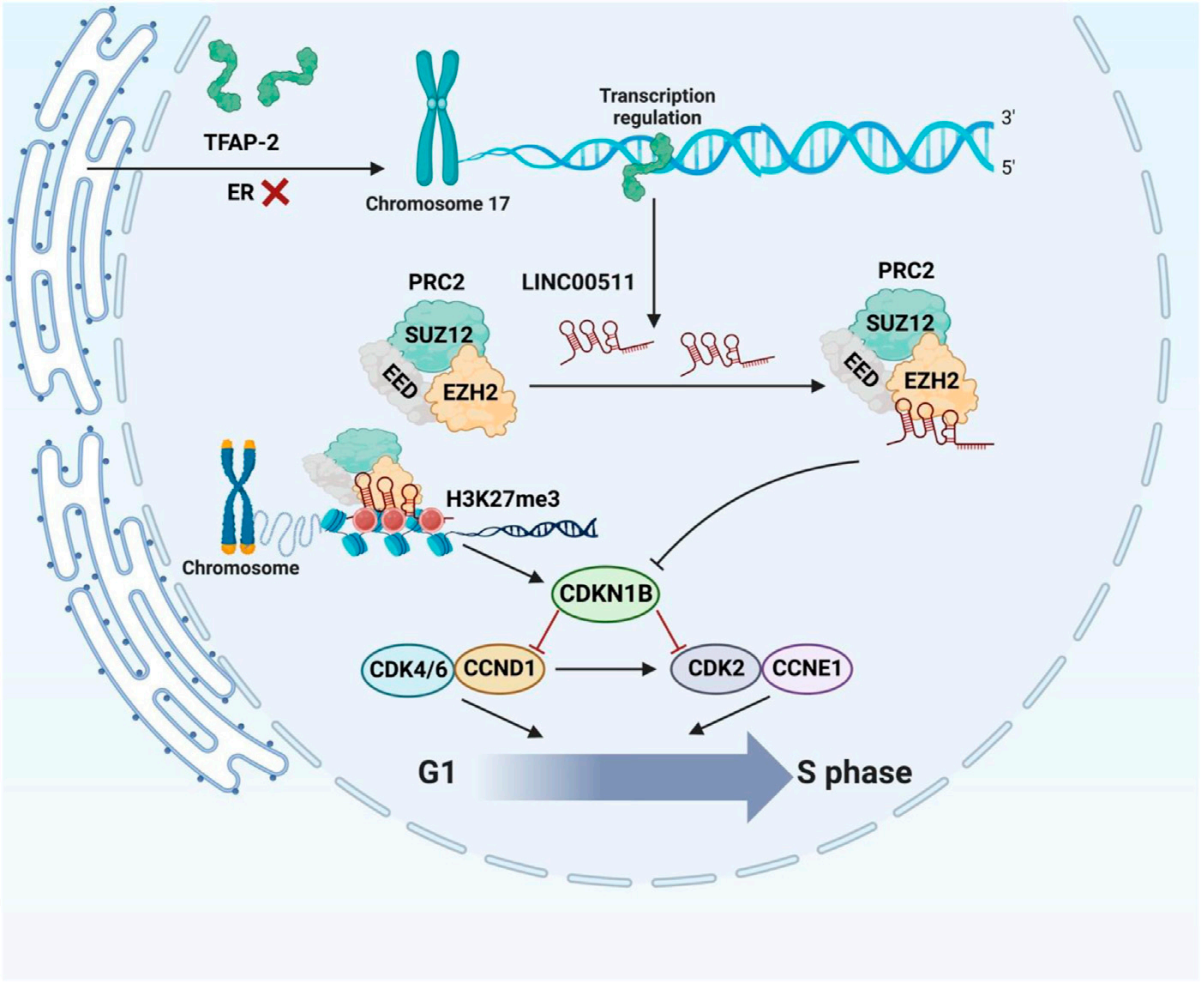
The carcinogenic role of LINC00511 in ER-negative tumorigenesis. ER insufficiency enhanced TFAP-2 activity at particular promoter regions, promoting LINC00511 expression. By interacting with EZH2 to attract PRC2 to regulate histone methylation, the ER-negative-associated LINC00511 repressed the expression of CDKN1B, assisting in the G1/S transition to maintain cellular growth.

Another study in patients with breast cancer has reported upregulation of LINC00511 and miR-301a-3p in patients’ blood parallel with downregulation of miR-185-3p (Mahmoud et al., 2021). Notably, LINC00511 expression has been increased in early stages of breast cancer. Area under the reciever operating characteristic curves of LINC00511, miR-185-3p, and miR-301a-3p has been superior to classical tumor markers indicating the diagnostic values of these transcripts as molecular biomarkers in liquid biopsy. Moreover, expression of LINC00511 has been correlated with lymph node metastasis and advanced tumor grades (Mahmoud et al., 2021). Additionally, the sponging effect of LINC00511 on miR-185 has been shown to be involved in breast cancer recurrence and radioresistance via regulation of STXBP4 expression (Liu et al., 2019). In breast cancer cells, LINC00511 expression induced by TFAP-2 expression and directly affected by ER deficiency at the transcriptional level. Through its interaction with EZH2, LINC00511 has been shown to encourage tumor development and suppress apoptosis (Figure 2) (Zhang et al., 2019). Table 1 summarizes the results of studies regarding the role of this lncRNA in breast cancer.

**TABLE 1:** Role of LINC00511 in breast cancer.

Cancer type	Expression/Role	Samples/Assessed cell lines	Pathways	Targets/Regulators	Function	Ref
Breast Cancer (BC)	Upregulated/Oncogene	39 cases of breast cancer/MCF-10A, MDA-MB-468, MDA-MB-231, MDA-MB-453, MCF-7	miR-185-3p/E2F1/Nanog axis	miR-185-3p/E2F1	LINC00511/miR-185-3p/E2F1/Nanog axis has a role in breast cancer stemness and malignancy	Lu et al. (2018)
	Upregulated/Oncogene	10 TNTPs/MDA-MB-231, MCF-7	LINC00511/miR-150/MMP13 axis	miR-150/MMP13	The LINC00511/miR-150/MMP13 axis could represent a novel treatment strategy for sufferers with breast cancer, because it is a breast cancer promoter	Shi et al. (2021)
	Upregulated/Oncogene	MDA-MB-231, MCF-7, T47D, MDA-MB-468, MCF-10a	-	MET, E2F2, TGFA, and WNT10A	Patients with breast cancer who express LINC00511 more than usual have a bad outcome. Breast cancer progression is facilitated by LINC00511	Liu et al. (2021a)
	Upregulated/Oncogene	21 TNTPs/MDA-MB-231, MCF-7, Hs-578T, T47D, MCF-10A	LINC00511/miR-29c/CDK6 axis	miR-29c/CDK6	Through controlling the miR-29c/CDK6 axis, LINC00511 lowering increased paclitaxel cytotoxicity in BC cells. A viable BC therapeutic option might be LINC005111	Zhu et al. (2019)
	Upregulated/Oncogene	25 patients and 25 control samples	LINC00511/miR-185-3p axis	miR-301a-3p	A more accurate diagnosis of BC may be made using serum LINC00511 and miR-301a-3p as prospective molecular indicators	Mahmoud et al. (2021)
	Upregulated/Oncogene	MDA-MB-231, MDA-MB-436	LINC00511/miR-185/STXBP4 axis	miR-185/STXBP4	Inhibition of LINC00511 reduces It has ability to bind competitively to miR-185, which boosts STXBP4 production and enhances radiation responsiveness in BC. A prospective treatment approach for boosting the prognosis of BC is the LINC00511/miR-185/STXBP4 axis	Liu et al. (2019)
	Upregulated/Oncogene	-	Apoptosis pathway	-	Through the suppression of antiapoptotic genes, LINC00511 deletion procedures using CRISPR/Cas9 improved the apoptosis of breast cancer cells	Azadbakht et al. (2022)
	Upregulated/Oncogene	MCF7, UACC-812, MDA-MB-231	-	EZH2 and CDKN1B	In ER-negative breast cancer, lncRNAs have a role in controlling the network of cell cycle regulation, and this has led to speculation that LINC00511 may be used as an anticancer treatment	Zhang et al. (2019)
	Upregulated/Oncogene	15 TNTPs	LINC00511/hsa-miR-573/GSDMC axis	miR-573/GSDMC	The most probable ncRNA-related mechanisms that drive GSDMC in BRCA are thought to be the LINC00511/hsa-miR-573 axis	Sun et al. (2022)
	Upregulated/Oncogene	7 TNTPs (HER-2-enriched)	-	-	A putative chemical indicator and viable treatment option for breast cancer of the HER-2-enriched subgroup is LINC00511	Yang et al. (2016)

### Gastric cancer

LINC00511 has been shown to promote progression of gstaric acnecr through regulating SOX4 expression and epigenetically suppressing PTEN to induce activity of PI3K/AKT pathway (Wang et al., 2021). Moreover, LINC00511 can promote growth of gastric tumors through acting as a molecular sponge for miR-124-3p and regulating expression of PDK4 (Sun et al., 2020a). miR-515-5p is another miRNA that is sponged by LINC00511 in gastric cancer cells leading to enhancemnet of proliferation and invasion of these cells (Wang et al., 2020a). Finally, miR-625-5p/NFIX (Chen et al., 2019a), miR-124-3p/EZH2 (Huang et al., 2020), miR-625-5p/STAT3 (Cui et al., 2021) and miR-29b/KDM2A (Zhao et al., 2020) are other molecular axes regulated by LINC00511 in gastric cancer. Table 2 shows the role of LINC00511 in gastric cancer.

**TABLE 2:** Role of LINC00511 in gastric cancer.

Cancer type	Expression/Role	Samples/Assessed cell lines	Pathways	Targets/Regulators	Function	Ref
Gastric Cancer (GC)	Upregulated/Oncogene	AGS, HGC‐27, ACP01, SNU‐1, Het‐1A	PI3K/AKT pathway	miR‐195‐5p/SOX4	After epigenetically suppressing PTEN to activate the PI3K/AKT pathway by engaging EZH2, SOX4-induced LINC00511 stimulated SOX4 via ceRNA pattern, promoting GC cell proliferation, migration and stemness while preventing GC cell death	Wang et al. (2021)
	Upregulated/Oncogene	MKN-45, BGC-823, HGC-27, MGC-803, GES-1	LINC00511/miR-124-3p/PDK4 axis	miR-124-3p/PDK4	By functioning as a ceRNA to control the miR-124-3p/PDK4 axis, which could be a viable therapeutic option for GC, LINC00511 encourages the tumor cell growth	Sun et al. (2020a)
	Upregulated/Oncogene	25 patients with gastric cancer/GES-1, AGS, SGC7901, BGC823, MKN45, MGC803	MAPK signaling pathway	miR-515-5p	By influencing miR-515-5p, LINC00511 can stimulate the expansion of tumor cells, suggesting that it might be a viable option for the creation of anti-cancer medications	Wang et al. (2020a)
	Upregulated/Oncogene	35 TNTPs/GES1, GC27, BGC823, MGC803, SGC7901	LINC00511/miR-625-5p/NFIX axis	miR-625-5p/NFIX	By targeting NFIX in GC cells, LINC00511 is a tumor activator to sponge miR-625-5p. Elimination of this lncRNA might be viewed as a treatment option for GC therapy	Chen et al. (2019a)
	Upregulated/Oncogene	80 patients with GC/GES-1, MGC, HGC, MKN25, MKN28	LINC00511/miR-124-3p/EZH2 pathway	miR-124-3p/EZH2 pathway	In sufferers with GC, LINC00511 is linked to a poor overall survival. It was predicted that LINC00511 would be a suitable target for treating human GC since it has a function in encouraging the cancerous cells growth	Huang et al. (2020)
	Upregulated/Oncogene	50 TNTPs/MKN28, BGC-823, MKN-45, MGC-803, SGC-7901, GES-1	STAT3 signal pathway	miR-625-5p/STAT3	By controlling miR-625-5p and STAT3, LINC00511 encourages GC cell growth, implying that LINC00511 has oncogenic capabilities that influence the formation of GC.	Cui et al. (2021)
	Upregulated/Oncogene	AGS, SGC7901	LINC00511/miR-29b/KDM2A axis	miR-29b/KDM2A axis	In GC, LINC00511 depletion boosted the apoptosis and hindered cell growth. It is possible to exploit the LINC00511/miR-29b/KDM2A axis as a viable treatment option for GC.	Zhao et al. (2020)

### Lung cancer

LINC00511 has ben shown to promote proliferation, invasive capacities, and migration of non-small cell lung cancer cells through regulating miR-625-5p/GSPT1 axis (Cheng et al., 2021). Moreover, LINC00511 can promote progression of this type of cancer through binding to EZH2 and LSD1 and decreasing expression levels of LATS2 and KLF2 (Zhu et al., 2019). Besides, this lncRNA has a role in induction of tumor recurrence in this type of cancer through sponging miR-98-5p and increasing expression of TGFBR1 (Li et al., 2022). LINC00511 can also induce resistance of lung cancer cells to cisplatin through sponging miR-625 and influencing expression of LRRC8E (Liu et al., 2022). Finally, this lncRNA exerts its oncogenic effects in lung cancer through binding to EZH2 and decaresing expression of p57 (Sun et al., 2016). Table 3 shows the role of LINC00511 in lung cancer.

**TABLE 3:** Role of LINC00511 in lung cancer.

Cancer type	Expression/Role	Samples/Assessed cell lines	Pathways	Targets/Regulators	Function	Ref
Lung Squamous Cell Carcinoma (LUSC)	Upregulated/Oncogene	SK-MES-1, H226, 16-HBE	LINC00511/miR-150-5p/TADA1 axis	miR-150-5p/TADA1	A plan for the therapeutic targeting of LINC00511 in LUSC should be developed, because it stimulates the advancement of LUSC.	Wu et al. (2020)
Lung Adenocarcinoma (LUAD)	Upregulated/Oncogene	45 LAC tissues and corresponding adjacent normal lung tissues/BEAS‐2B, H1299, A549	PKM2 signaling pathway	miR-625-5p/PKM2	By influencing miR-625-5p/PKM2, LINC00511 was found to be engaged in the evolution of LAC, demonstrating that LINC00511/miR-625-5p/PKM2 may represent interesting treatment targets for LUAC.	Xue and Zhang (2020)
	Upregulated/Oncogene	40 TNTPs/A549, Calu-3, BEAS-2B, DV-90, PC-9	LINC00511/miR-195-5p/GCNT3 axis	miR-195-5p/GCNT3	By suppressing miR-195-5p, LINC00511 reduction encourages GCNT3 production, and as a result, supports the malignant growth of LUAD.	Zhang et al. (2022)
	Upregulated/Oncogene	A549/DDP, 16HBE	LINC00511/miR-182/BIRC5 axis	miR-182-3p/BIRC5	After injection of cisplatin (DDP), LINC00511 silencing prevents the growth of A549/DDP cells into tumors. In order to understand the mechanism of gained DDP tolerance, this research offers a unique LINC00511/miR-182/BIRC5 model	Zhu et al. (2022)
	Upregulated/Oncogene	35 TNTPs/A549, PC9, BEAS-2B	Linc00511/miR-126-5p/miR-218-5p/COL1A1 axis, PIK3/AKT pathway	miR-126-5p, miR-218-5p/COL1A1	By targeting miR-126-5p and miR-218-5p, LINC00511 influences COL1A1 production, encouraging cancerous cell growth and motility. LINC00511 could be a viable treatment approach for LUAD.	Wang et al. (2022)

### Other types of cancers

Over-expression of LINC00511 has been reported in a variety of cancers including colorectal, pancreatic, liver, thyroid and other types of cancers (Table 4).

**TABLE 4:** Dysregulation of LINC00511 in different cancers (TNTP: tumor and non-tumor pairs of tissues).

Cancer type	Expression/Role	Samples/Assessed cell lines	Pathways	Targets/Regulators	Function	Ref
Hepatocellular Carcinoma (HCC)	Upregulated/Oncogene	Huh7, Hep3B	-	RAB27B	There seems to be a link between the creation of invadopodia and the generation of exosomes and LINC00511 dysregulation. In HCC, LINC00511 could be a treatment option	Peng et al. (2021)
	Upregulated/Oncogene	SMCC7721, HepG2, Huh7, Hep3B	LINC00511/miR195/EYA1 axis	miR195/EYA1	A prospective therapeutic option for the detection of HCC has been provided by LINC00511 that interacted with EYA1 to accelerate HCC formation through miR-195	Hu et al. (2020a)
	Upregulated/Oncogene	127 TNTPs/LO2, Hep3B, HepG2, SMMC-7721, MHCC97H, Huh7, HCCLM3	-	miR-424	LINC00511 may have a significant impact on how HCC develops, and that it will also act as a viable prognostic and therapeutic target.	Wang et al. (2019a)
Liver Hepatocellular Carcinoma (LIHC)	Upregulated/Oncogene	LO2, MHCC-97H, Huh7, HCC-LM3, Hep3B, MHCC-97L, Huh6	-	miRNA-29c	Through boosting tumor cell proliferative ability, LINC00511 worsens LIHC’s development. A predictive marker for LIHC could be LINC00511	Liu et al. (2019)
Osteosarcoma (OS)	Upregulated/Oncogene	24 TNTPs/SW1353, U2OS	LINC00511/miR-185-3p/E2F1 axis	miR-185-3p/E2F1	As an oncogenic RNA, LINC00511 leads to the formation and spread of tumor cells. The LINC00511/miR-185-3p/E2F1 axis may be extremely important for the onset of osteosarcoma	Xu et al. (2020)
	Upregulated/Oncogene	30 TNTPs s/(MG‐63, Saos‐2, U2OS, HOS, NHOst	-	miR‐765	Via substantially controlling the production of miR-765, aberrant transcription of LINC00511 boosted osteosarcoma cell tumorigenesis and motility	Yan et al. (2018)
	Downregulated/Tumor suppressor gene	45 patients with osteosarcoma/hFOB1.19, MG-63, U‐2OS, Saos‐2, HOS	-	-	Elevated amounts of LINC00511 slow the growth of tumors. LINC00511 could be a new indicator and prospective osteosarcoma treatment approach	Qiao et al. (2020)
	Upregulated/Oncogene	10 TNTPs/hFOB 1.19, MG-63, HOS, Saos-2, 143B	LINC00511/miRNA- 618/MAEL axis	miRNA- 618/MAEL	OS cell growth and malignancy were both hindered by lower LINC00511 production. It could be a viable biomarker for more exploration on the treatment of OS.	Guo et al. (2019)
T-cell Acute Lymphoblastic Leukemia (T-ALL)	Upregulated/Oncogene	Blood samples of 35 T-ALL patients and 30 normal controls/HPB-ALL, TALL-1, ALL-SIL, CUTLL1, PBMC	LINC00511/miR-195-5p/LRRK1 axis	miR-195-5p/LRRK1	Through the miR-195-5p/LRRK1 axis, LINC00511 accelerated the evolution of T-ALL, pointing a possible therapeutic hint for the T-ALL sufferers	Li et al. (2020)
Glioma	Upregulated/Oncogene	HEB, NHA, T98 G cells (CRL-1690), A172 cells (CRL-1620), LN229 cells (CRL-2611), U-87MG cells (HTB-14IG)	LINC00511/miR-15a-5p/AEBP1 axis	miR-15a-5p/AEBP1	Glioma formation can be slowed down by LINC00511 knockdown. Additionally, via the miR-15a-5p/AEBP1 axis, the process of LINC00511 influences the onset of glioma	Liu et al. (2021b)
	Upregulated/Oncogene	U87, U251, SHG44, A172, NHA	SP1/LINC00511/miR‐124‐3p/CCND2 axis	miR‐124‐3p/CCND2	The transcription factor SP1 served as the inspiration for the overexpression of LINC00511 in glioma cells. Additionally, the upregulation of LINC00511 competitively sponges the miR-124-3p, driving the production of CCND2 and the cyclin D2 protein produced by this gene, which may hold significant potential for glioma therapies	Li et al. (2019)
Papillary Thyroid Carcinoma (PTC)	Upregulated/Oncogene	41 TNTPs/B-CPAP, KTC-1, KTC-1	-	CDKs and EZH2	As an oncogene in PTC, LINC00511 promotes proliferation through CDKs. It will serve as a fundamental therapeutic target for PTC.	Xiang et al. (2020)
Colorectal Cancer (CRC)	Upregulated/Oncogene	85 CRC tissues and adjacent normal tissues/HCT116, HT-29, LoVo, SW480, SW620, NCM460	HNF4α/LINC00511/IL24 axis	HNF4α and IL24	The proliferative, spreading and aggressive features of CRC cells are lowered by LINC00511 reduction, that eventually reduces tumorigenicity of CRC.	Lu et al. (2021)
	Upregulated/Oncogene	120 TNTPs/SW480, SW620, HCT16, HT29, NCM460	LINC00511-mediated microRNA (miR)-625-5p/WEE1 axis	miRNA-625-5p/WEE1	By suppressing WEE1 and restoring miR-625-5p, downregulated LINC00511 prevents the carcinogenesis of CC, establishing a fundamental standard for CC-targeted treatment	Qian et al. (2022)
	Upregulated/Oncogene	12 TNTPs/HT-29, HCT8, HCE8693, SW620, NCM460	LINC00511/miR-29c-3p/NFIA axis	miR-29c-3p/NFIA	By inhibiting the LINC00511/miR-29c-3p/NFIA axis, LINC00511 assisted in the onset of CRC, proposing that LINC00511 could be a viable therapeutic target.	Hu et al. (2020b)
Glioblastoma (GBM)	Upregulated/Oncogene	160 TNTPs/U87, A172, U138, U251, U373, LN‐18, T98G, Human HEK293T	Wnt/β-catenin signaling	miR‐126‐5p	In GBM cells, LINC00511 controlled Wnt/Catenin stimulation by functioning as a molecular sponge for miR-126-5p. It stated that LINC00511 could operate as a marker for the treatment of GBM.	Wang et al. (2021)
	Upregulated/Oncogene	36 GBM tissues and 8 non-tumour brain tissues (NBT)/U87, LN229, U251, A172, 293T	LINC00511/miR‐524‐5p/YB1/ZEB1 axis	miR‐524‐5p/YB1	By boosting EMT, the LINC00511/miR524-5p/YB1/ZEB1 positive feedback loop might encourage GBM cell motility and infiltration. LINC00511 could be a viable therapeutic target for GBM sufferers	Du et al. (2020)
Esophageal Cancer (ECa)	Upregulated/Oncogene	-	-	miR-150-5p	By attaching to miR-150-5p and sponging this miRNA, LINC00511 controls the creation of cancerous cells and, could be a treatment option for ECa	Sun et al. (2020a)
Cervical Cancer (CC)	Upregulated/Oncogene	-	LINC00511/miR-497-5p/MAPK1 axis	miR-497-5p/MAPK1	Overexpression of LINC00511 boosted CC cell growth, motility and infiltration, whereas LINC00511 reduction had the opposite effects	Lu et al. (2022)
	Upregulated/Oncogene	92 cervical cancer tissues and 40 adjacent normal tissues/SiHa, HeLa, C33A, Caski, Ect1/E6E7	-	-	In cervical cancer sufferers, high LINC00511 transcription is linked to clinical deterioration. The growth, motility and infiltration of tumor cells are restricted by LINC00511 suppression	Yu et al. (2019)
	Upregulated/Oncogene	40 TNTPs/HT29, LOVO, SW620, SW480	LINC00511/miR-153-5p/HIF-1α axis	miR-153-5p/HIF-1α	A crucial part of the CRC carcinogenesis is played by LINC00511. HIF-1α/LINC00511/miR-153-5p might be used as a therapeutic target in CRC.	Sun et al. (2020b)
	Upregulated/Oncogene	19 TNTPs/SiHa, CaSki, C33A, HUCEC	LINC00511/miR-324-5p/DRAM1 axis	miR-324-5p/DRAM1	The miR-324-5p/DRAM1 axis is regulated by LINC00511, acting as a ceRNA which, promotes the progression of both HPV-negative and HPV-positive cervical cancer	Zhang et al. (2020)
	Upregulated/Oncogene	84 CC patients/PTX-resistant Hela/PTX	-	-	Suppression of LINC00511 may lessen CC cell motility and infiltration as well as paclitaxel tolerance, and may boost cell death in CC cells. LINC00511 suppression offers CC a brand-new treatment option	Mao et al. (2019)
	Upregulated/Oncogene	47 TNTPs/SiHa, CaSki, C33A, ME180, HeLa, NCECs	-	RXRA or PLD1	In CC, LINC00511 promotes the production of PLD1, which is controlled by RXRA. It could be a viable indicator for the therapy of CC.	Shi et al. (2020)
Cervical Squamous Carcinoma (CESC)	Upregulated/Oncogene	115 cases of CESC, 79 cases of cervical intraepithelial neoplasia and 101 healthy controls	-	-	A diagnostic model consisting of CCAT2 and LINC01133 has exhibited significant clinical utility for the identification of cervical intraepithelial neoplasia in both healthy individuals and patients. The serum concentrations of CCAT2, LINC01133 and LINC00511 could be used as effective non-invasive indicators for the diagnosis of CESC.	Wang et al. (2020b)
Clear Cell Renal Cell Carcinoma (ccRCC)	Upregulated/Oncogene	49 TNTPs/HK-2, A498, 786-O, ACHN, Caki-2	LINC00511/miR-625/CCND1 pathway	miRNA-625/cyclin D1	As a ceRNA, LINC00511 controls the transcription of CCND1 in ccRCC via sponging miR-625. As a result, the LINC00511/miR-625/CCND1 pathway could offer ccrCC patients a prospective treatment approach	Deng et al. (2019)
Bladder Cancer (BcA)	Upregulated/Oncogene	47 TNTPs/TCCSUP, SW780	LINC00511/miR-143-3p/PCMT1 axis	miR-143-3p/PCMT1	By suppressing the production of miR-143-3p and promoting the production of PCMT1, LINC00511s molecular mechanism may prevent bladder cancer cells from proliferating and invading. A novel target for bladder cancer treatment may be offered by LINC00511	Dong et al. (2021)
	Upregulated/Oncogene	45 TNTPs/SV-HUC-1, BIU87, T24, 5637	Wnt/β-catenin signaling pathway	miR-15a-3p	By inhibiting the Wnt/β-catenin signaling pathway activity, LINC00511 suppression decreases bladder cancer cells ability to proliferate and increases their likelihood of dying. LINC00511 could also be a putative bladder cancer indicator and possible therapeutic target.	Li et al. (2018)
Tongue Squamous Cell Carcinoma (TSCC)	Upregulated/Oncogene	Tca-8113	LINC00511/miR-765/LAMC2 axis	miR-765/LAMC2	By sponging miR-765 with ceRNA, LINC00511 increases the production of LAMC2. the ceRNA regulation network contributes to new knowledge about the pathophysiology of TSCC and given information on how to take advantage of the emerging area of lncRNA-directed treatment for TSCC.	Ding et al. (2018)
Non-small Cell Lung Cancer (NSCLC)	Upregulated/Oncogene	67 patients/16HBE, A549, NCIH1299, NCIH1650, NCIH 1975, NCIH460	LINC00511/miR-625-5p/GSPT1 axis	miR-625-5p/GSPT1	By targeting miR-625-5p/GSPT1, LINC00511 boosts NSCLC cell growth, infiltration and motility. LINC00511 is a possible diagnostic indicator and treatment option for NSCLC.	Cheng et al. (2021)
	Upregulated/Oncogene	57 TNTPs	-	EZH2 and LSD1/LATS2 and KLF2	Apoptosis is triggered in NSCLC cells when LINC00511 is knocked down, although this reduces the capability of the cells to spread and infiltrate	Zhu et al. (2019)
	Upregulated/Oncogene	62 stage I NSCLC patients and the age- and gender-matched healthy controls	LINC00511/miR-98-5p/TGFBR1 axis	miR-98-5p/TGFBR1	In NSCLC, LINC00511 quantities were raised, which may contribute to distant postoperative recurrence of NSCLC and enhance NSCLC cell growth, motility and penetration by targeting and controlling the miR-98-5p/TGFBR1 axis	Li et al. (2022)
	Upregulated/Oncogene	40 TNTPs/A549, H522, A549/DDP, H522/DDP, BEAS-2B	LINC00511/miR-625/LRRC8E pathway	miR-625/LRRC8E	LINC00511 enhanced LRRC8E production by downregulating miR-625 to boost DDP tolerance in NSCLC. A viable therapeutic target to reduce DDP tolerance in NSCLC is LINC00511	Liu et al. (2022)
	Upregulated/Oncogene	124 TNTPs/A549, SK-MES-1, H1299, 95D, H460, H520, H1975, H157, SK-LU-1, SPC-A-1, 16HBE	-	EZH2 and p57	In NSCLC, LINC00511 is clinically, physiologically and molecularly oncogenic	Sun et al. (2016)
Pancreatic Cancer (PC)	Upregulated/Oncogene	91 TNTPs/BxPC-3, CFPAC-1, PANC-1, SW 1990, MIAPaCa-2, HPDE6-C7	LINC00511/miR-370-5p/p21 Axis	miR-370-5p/p21, Snail, and ZEB1	The LINC00511/miR-370-5p/p21 promoter region axis was responsible for the suppressive impact of DET (deoxyelephantopin) on the growth and spread of PC cells	Ji et al. (2022)
Pancreatic Ductal Adenocarcinoma (PDAC)	Upregulated/Oncogene	140 TNTPs, PANC‐1, MIA PaCa‐2, Capan‐2, SW 1990, ASPC‐1, BxPC‐3, HPDE6	LINC005/hsa‐miR29b‐3p/VEGFA axis	miR‐29b‐3p/VEGFA	The etiology of PDAC is profoundly influenced by the new lncRNA LINC00511. LINC00511 is a unique predictive indicator that can forecast the clinical outcomes of PDAC sufferers following surgery and could be used as a treatment option for PDAC.	Zhao et al. (2018)
Thyroid Carcinoma (TC)	Upregulated/Oncogene	TPC-1, BCPAP, IHH-4, Nthy-ori 3–1	JAK2/STAT3 signaling pathway and LINC00511/TAF1/JAK2 axis	TAF1 and JAK2	Through TAF1-mediated JAK2/STAT3 signaling, enhanced expression of LINC00511 increased the radiosenitivity of TC cells. The potential biomarker function of LINC00511 in the management of TC was shown by the recent research	Chen et al. (2019b)
Ovarian Cancer (OC)	Upregulated/Oncogene	CAOV3, OVCAR3, SKOV3, UWB1.289	-	miR-424-5p and miR-370-5p/ESR1	With the suppression of cell death, upregulated LINC00511 boosted the vitality, motility and penetration of CAOV3 cells. The disruption of miR-424-5p and miR-370-5p is likely responsible for these actions that promote malignancy	Wang et al. (2019b)
	Upregulated/Oncogene	SKOV3, SNU840	-	EZH2 and P21	The growth of OC cells is slowed by LINC00511 silencing. This information may offer a valuable lncRNA as a predictive indicator and possible treatment option	Deng et al. (2019)

### Transcriptional regulation of LINC00511

Investigations in the Hormonizome database (Rouillard et al., 2016) indicated that 18 transcription factors (CTCF, EP300, ESR1, EZH2, FOXA1, GATA3, H2AFZ, MAX, MYC, NFIC, NR2F2, NR3C1, POLR2A, RAD21, TCF12, TEAD4, YY1, and ZBTB7A) possibly bind to the promoter of LINC00511 gene based on ChIP-seq data from the ENCODE Transcription Factor Target dataset.

### LINC00511 related pathways and functions.

Based on lncHUB database (https://maayanlab.cloud/lnchub/), 10 KEGG pathways with the highest Z-score in which LINC00511 is predicted to be involved include glycosphingolipid biosynthesis, bacterial invasion of epithelial cells, basal cell carcinoma, central carbon metabolism in cancer, notch signaling pathway, RNA polymerase, DNA replication, cell cycle, bladder cancer and mismatch repair. Additionally, 10 gene ontology (GO) terms with the highest Z-score that are associated with LINC00511 include regulation of hydrogen peroxide-induced cell death (GO:1903205), negative regulation of response to reactive oxygen species (GO:1901032), protein heterotetramerization (GO:0051290), viral release from host cell (GO:0019076), exit from host cell (GO:0035891), signal complex assembly (GO:0007172), regulation of striated muscle tissue development (GO:0016202), regulation of myelination (GO:0031641), positive regulation of kidney development (GO:0090184) and regulation of proteolysis (GO:0030162). Also, IGSF11, PHLPP1, CPOX, SOX2, PACC1, SOX21, TMPRSS5, HEY1, MARCKS, KCTD5, OLIG2, ATAT1, CDK5R1, BCAN and BAALC are 15 genes with the highest Z-score predicted to be co-expressed with LINC00511.

### LINC00511 interactions with miRNAs and other molecules

Based on RNAInter (RNA Interactome Database) (Kang et al., 2022), LINC00511 has interactions with hsa-miR-29b-3p, hsa-miR-765, hsa-mir-150, miR-1231, TFAP2A-AS2, hsa-miR-185-3p, hsa-miR-29b-1-5p, hsa-miR-29c-3p, RAD51-AS1 and EZH2 with score ≥0.1. Also, based on LncRNA2Target v3.0 (Cheng et al., 2019), LINC00511 interactions with miRNAs has been showed in Table 5.

**TABLE 5:** LINC00511 interactions with miRNAs.

Target gene	LncRNA experiment	Tissue	Cell line	Disease state	Reference
MIR29B1	Luciferase reporter assays, RNA immunoprecipitation	Pancreatic cancer	PANC-1, MIA PaCa-2, Capan-2, SW 1990, ASPC-1, BxPC-3	Pancreatic cancer	Zhao et al. (2018)
MIR765	Dual luciferase reporter assays	NA	Tca-8113	Tongue squamous cell carcinoma	Ding et al. (2018)
MIR524	Dual-luciferase gene reporter assay, RNA immunoprecipitation (RIP)	Brain	U87, LN229, U251, A172, 293T	Glioblastoma Multiforme	Du et al. (2020)
miR-124-3p	Luciferase assay	GC tissue	GES-1, MGC, HGC, MKN25, MKN28	Gastric cancer	Huang et al. (2020)
MIR150	Luciferase reporter assay	Breast	MDA-MB-231, MCF-7	Breast Cancer	Shi et al. (2021)

## Impact of LINC00511 dysregulation on clinical outcome of patients with cancers

**FIGURE 3:**
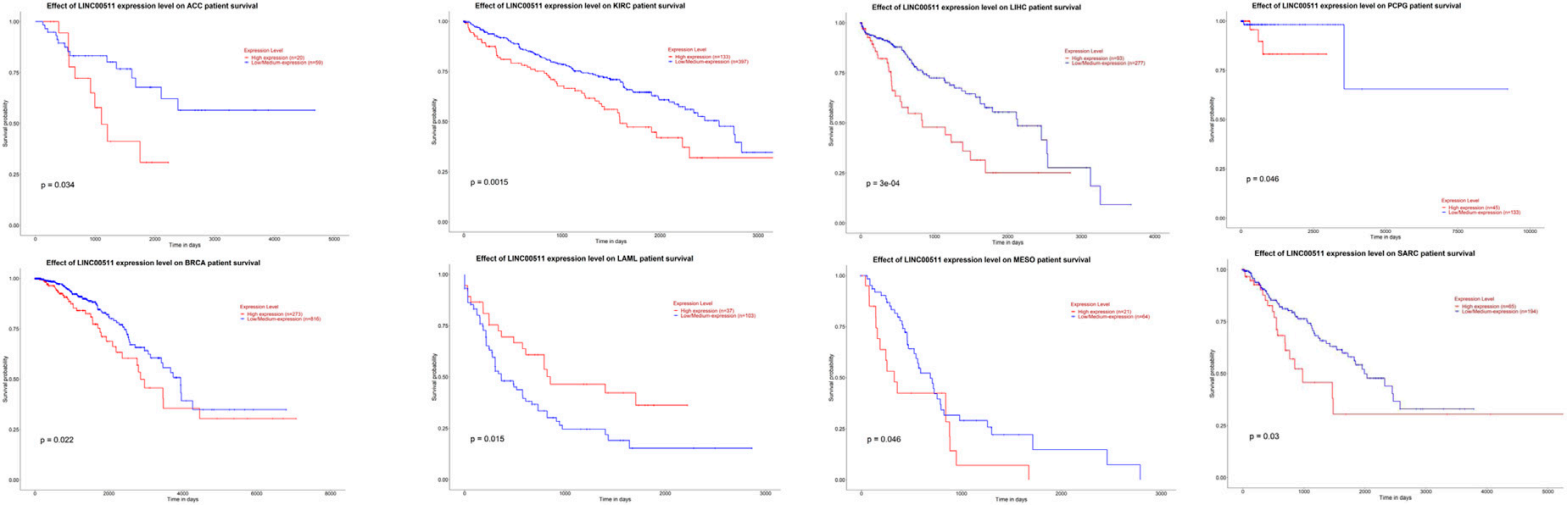
The impact of LINC00511 on survival of patients with different cancers.

We investigated the survival rate caused by LINC00511 in different cancers using ualcan database (Chandrashekar et al., 2017). This database performs survival analysis using TCGA data. The difference was statistically significant with a log-rank *p*-value less than 0.05. As a result, LINC00511 has an effect on the survival rate of patients with adrenocortical carcinoma (ACC), breast invasive carcinoma (BRCA), kidney renal clear cell carcinoma (KIRC), acute myeloid leukemia (LAML), liver hepatocellular carcinoma (LIHC), Mesothelioma (MESO), pheochromocytoma and paraganglioma (PCPG) and sarcoma (SARC) (Figure 3). While in patients with LAML, overexpression of this lncRNA is associated with better clinical outcome, in other types of cancers, its upregulation is associated with lower survival.

## Discussion

LINC00511 is a lincRNA being over-expressed in a variety of human tumors and cancer cell lines. Except for a single study in osteosarcoma (Qiao et al., 2020), other studies in this type of cancer and other cancers have reported upregulation of LINC00511 in tumoral tissues compared with their non-tumoral counterparts. Dysregulation of LINC00511 affects cancer pathogenesis through increasing cell proliferation and inhibiting cell apoptosis. It can also increase activity of several cancer-promoting signaling pathways.

A previous meta-analysis has reported association between over-expression of LINC00511 and poor prognosis in different cancers in terms of overall, progression-free or relapse-free survival times (Agbana et al., 2020). Moreover, upregulation of this lncRNA has been associated with larger tumor size, recurrent disorder and metastasis to lymph nodes or distant organs (Agbana et al., 2020).

Mechanistically, LINC00511 can act as a molecular sponge for a variety of miRNAs regulating their targets. miR-185-3p/E2F1, miR-150/MMP13, miR-29c/CDK6, miR-185/STXBP4, miR-573/GSDMC, miR‐195‐5p/SOX4, miR-124-3p/PDK4, miR-625-5p/NFIX, miR-124-3p/EZH2, miR-625-5p/STAT3, miR-29b/KDM2A, miR-195/EYA1, miR-185-3p/E2F1, miRNA-618/MAEL, miR-195-5p/LRRK1, miR-15a-5p/AEBP1, miR‐124‐3p/CCND2, miRNA-625-5p/WEE1, miR-29c-3p/NFIA, miR-150-5p/TADA1, miR‐524‐5p/YB1, miR-625-5p/PKM2, miR-195-5p/GCNT3, miR-182-3p/BIRC5, miR-218-5p/COL1A1, miR-497-5p/MAPK1, miR-153-5p/HIF-1α, miR-324-5p/DRAM1, miR-625/cyclin D1, miR-143-3p/PCMT1, miR-765/LAMC2, miR-625-5p/GSPT1, miR-98-5p/TGFBR1, miR-625/LRRC8E, miR-370-5p/p21, miR‐29b‐3p/VEGFA and miR-370-5p/ESR1 are examples of miRNA/mRNA axes that are regulated by LINC00511. Molecular axes being regulated by LINC00511 in more than one type of cancer represent better targets for design of anti-cancer therapies since they can be applied in a wider range of malignancies. Therefore, identification of the impact of above-mentioned molecular axes in the progression of different types of cancer is an important step in design of novel therapeutics.

LINC00511 can induce stemness in cancers and facilitate tumor progression and metastasis (Lu et al., 2018). Therefore, LINC00511-modifying modalities can be used as possible strategies for defeating cancer metastasis.

The prognostic role of over-expression of LINC00511 in different cancers has been evaluated thoroughly by various research groups indicating its important effects on survival of affected individuals. Future studies should assess its expression in biofluids to provide a non-invasive route for cancer diagnosis and patients’ follow-up.
